# Management of the Clinical and Academic Mission in an Urban Otolaryngology Department During the COVID-19 Global Crisis

**DOI:** 10.1177/0194599820929613

**Published:** 2020-05-19

**Authors:** Pete S. Batra, Phillip S. LoSavio, Elias Michaelides, Peter C. Revenaugh, Bobby A. Tajudeen, Samer Al-khudari, Inna Husain, Peter Papagiannopoulos, Ryan Smith, Kerstin M. Stenson, R. Mark Wiet

**Affiliations:** 1 Department of Otorhinolaryngology–Head and Neck Surgery, Rush University Medical Center, Chicago, Illinois, USA

**Keywords:** COVID-19, novel coronavirus, pandemic, otolaryngology, telehealth, tracheostomy

## Abstract

**Objective:**

The objective of this study was to assess the strategic changes implemented in the departmental mission to continue safe delivery of otolaryngology care and to support the broader institutional mission during the COVID-19 pandemic response.

**Study Design:**

Retrospective assessment was performed to the response and management strategy developed to transform the clinical and academic enterprise.

**Setting:**

Large urban tertiary care referral center.

**Results:**

The departmental structure was reorganized along new clinical teams to effectively meet the system directives for provision of otolaryngology care and support for inpatient cases of COVID-19. A surge deployment schedule was developed to assist frontline colleagues with clinical support as needed. Outpatient otolaryngology was consolidated across the system with conversion of the majority of visits to telehealth. Operative procedures were prioritized to ensure throughput for emergent and time-critical urgent procedures. A tracheostomy protocol was developed to guide management of emergent and elective airways. Educational and research efforts were redirected to focus on otolaryngology care in the clinical context of the COVID-19 crisis.

**Conclusion:**

Emergence of the COVID-19 global health crisis has challenged delivery of otolaryngology care in an unparalleled manner. The concerns for preserving health of the workforce while ethically addressing patient career needs in a timely manner has created significant dilemmas. A proactive, thoughtful approach that reorganizes the overall departmental effort through provider and staff engagement can facilitate the ability to meet the needs of otolaryngology patients and to support the greater institutional mission to combat the pandemic.

Coronavirus is a single-stranded RNA virus that has the potential to cause a spectrum of clinical symptoms, ranging from the common cold to more severe lower respiratory symptoms, including pneumonia, respiratory failure, and death.^1^ Typically, these viruses are found in animals worldwide, with few instances of human disease. The World Health Organization used the term 2019 novel coronavirus (COVID-19) to refer to the December 2019 outbreak in Wuhan, China, that affected the lower respiratory tract of patients and resulted in pneumonia.^2^ The reference name for the virus resulting in this disease has been termed severe acute respiratory syndrome coronavirus 2 (SARS-CoV-2). Given the rapid spread of COVID-19 globally over a matter of 2 months, the World Health Organization dubbed this a pandemic on March 11, 2020.^3^ As of April 19, 2020, the disease had spread to 185 countries, with 2,394,291 reported cases worldwide and 164,937 deaths.^4^

After the initial clustering of cases in China and subsequently Europe, the United States now sits in the epicenter of the pandemic. On April 19, 2020, the United States had the most cases in the world with 722,761, including 33,903 attributed deaths.^4^ As COVID-19 has reached the shores of our country, the impact of the evolving pandemic to otolaryngology has been profound. Within a matter of a few weeks, the specialty has seen a drastic decline in outpatient clinic volumes and the cessation of elective surgical procedures. Moreover, given the invasive nature of our diagnostic and therapeutic procedures, otolaryngologists have been deemed high risk for viral exposure, despite not being a traditional frontline specialty in battling the disease. Per anecdotal experience, colleagues in China, Italy, and Iran have reported high rates of COVID-19 transmissions to otolaryngologists, including deaths.

This article details the efforts of the otorhinolaryngology department at a large tertiary care referral center in Chicago that was designed to handle a pandemic. We hope to disseminate the strategies employed and lessons learned to continue to meet the tripartite otolaryngology mission under difficult circumstances and to support the greater mission of the organization to focus on managing cases of COVID-19.

## Setting

Rush University Medical Center is a 664-bed tertiary care referral hospital in the near west side of Chicago. The new hospital that opened in 2012 was designed with the capability to treat patients in particular crisis settings, such as a bioterrorism attack or a pandemic. The facility is 1 of 35 federally designated hospitals for treating infectious diseases. It houses 40 negative-pressure rooms to control airflow to entire sections of the building to prevent cross-contamination by airborne pathogens and the ability to expand bed capacity at short notice to manage a surge of potentially infected patients. The otorhinolaryngology department is composed of 14 full-time and 3 part-time otolaryngology faculty, 2 part-time oral maxillofacial surgeons, 9 audiologists, 7 ENT residents (ear, nose, and throat), and 4 advanced practice providers.

## Methods

Retrospective assessment of the management strategy of the Department of Otorhinolaryngology–Head and Neck Surgery at Rush University Medical Center to the evolving pandemic was conducted through review of daily institutional briefings, weekly departmental briefings, email communications from institution and department, and institutional and departmental written policies. The study was exempt from approval by the Rush University Medical Center Institutional Review Board.

## Results

### Institutional Response

Rush University Medical Center activated resources in late February to prepare for COVID-19, though an initial clustering of cases in the greater Chicago area was not seen until March 10, 2020.^5^ It is important to note that on April 16, 2020, the Illinois Department of Public Health notified the State Emergency Operations Center that hospitals may need to implement crisis standards of care pursuant to their disaster response plans. The changes in the hospital incident command structure were implemented 6 weeks prior to the invocation of crisis standards of care; thus, this represents a proactive emergency response to the looming crisis. Strategic institutional directives included development of a centralized command center, complete reorganization of the emergency department (ED) to triage patients with COVID-19, and implementation of the surge plan to handle floor and intensive care unit (ICU) cases.

A central COVID-19 hospital command center was activated with a team of 90 key hospital clinical and nonclinical leaders to coordinate the overall effort across the system of 3 hospitals and affiliated regional sites. Twice-daily hour-long briefings were initiated to review the caseload of system COVID-19 cases and system readiness in regard to personnel and resources, as well as local, regional, and national infection trends. The institution started daily system updates to apprise all employees on case numbers and related critical updates.

The ED was converted to handle coronavirus cases to mitigate risks to providers and patients in the direct line of initial exposure. The ambulance bay was converted into a COVID-19 triage center to manage any patients exhibiting coronavirus-like symptoms. Potentially infected patients were assessed in private screening tents for vitals, medical history, and COVID-19 testing. Any potential cases requiring admission to the general floor or ICU were directed to the 20-bed ED pod with private negative-pressure rooms, which were cordoned from the non–COVID-19 cases.

A separate coronavirus clinic in a stand-alone building, a telehealth provider pool, and a drive-through testing site for COVID-19–related care and testing were also implemented early to mitigate ED strain and potential staff and patient exposures. Testing capabilities were quickly increased through development of in-house testing as well as other private and institutional partnerships. Testing triage and indications were reviewed daily and expanded as testing capacity increased gradually over the course of the month. The introduction of point-of-care (POC) testing greatly expanded the capabilities by mid-April, especially in the ambulatory and operative setting. As of April 28, 16,223 patients have been screened across the Rush system since the beginning of the COVID-19 outbreak, with 4243 (26.2%) testing positive.

A surge capacity plan was implemented on March 2 to ensure adequate floor and ICU bed availability with the evolution of the pandemic. Through optimization of physical spaces and opening patient care units in nontraditional areas, such as postanesthesia care units, the hospital increased additional beds by 73% for critical care patients and by 58% for noncritical care patients. The total surge maximum capacity included 142 ICU and 460 non-ICU dedicated COVID-19 beds. This enhanced ability to treating patients with COVID-19 led to a significant increase in requests for transfers across the greater Chicago area. The organization has worked with hospitals across the region to selectively accept referrals where expertise and resources at Rush could be leveraged to handle difficult cases and to positively affect patient outcomes. However, these transfers had to be carefully balanced to preserve an appropriate level of capacity for incoming emergencies and non–COVID-19 cases within the system. As of April 8, 2020, Rush has accounted for 18% of ICU COVID-19 beds and 68% of vented ICU cases in the city of Chicago.

### Departmental Reorganization

The entire otolaryngology department was reorganized into a 2-team structure, with each team being composed of 7 full-time ENT faculty, 1 oral maxillofacial surgeon attending, 3 residents, and 1 inpatient advanced practice provider (**Table 1**). Call responsibilities were handled by each team, alternating every other day, with a primary faculty member on call and the remaining team members providing backup as needed for subspecialty consults and emergent cases. A single pager number was assigned to the rotating attendings on call, and sign-out was performed attending to attending each morning to maintain continuity of care on the service. This strategy would mitigate the need to have 1 individual on call for a long stretch of days. Furthermore, this would allow 2 fully staffed subspecialty services to work clinically apart to ensure continuity of services and redundancy within the department, in case there was any viral exposure to the providers. The lead team would also provide coverage for surge capacity to the ED, inpatient floors, and ICUs as needed to support frontline providers. Communication was strengthened across the department to ensure alignment among faculty, residents, administrators, and staff. Weekly web-based faculty meetings were implemented in addition to regular email communications from the chair, medical director, program director, and practice administrator.

**Table 1.** Departmental Reorganization Structure to Manage the COVID-19 Crisis.

Team A	Team B
Department chair and lead coordinator	
Team leader 1	Team leader 2
Faculty	
Rhinology 1	Rhinology 2
Head and neck 1	Head and neck 2
Otology/neurotology 1	Otology/neurotology 2
Facial plastics 1	Facial plastics 2
General main campus 1	General main campus 2
General off-site 1	General off-site 2
General/peds oto 1	General/peds oto 2
Oral surgery 1	Oral surgery 2
Residents/APPs	
Senior resident 1	Senior resident 2
Junior resident 1	Junior resident 2
Junior resident 1	Junior resident 2
Inpatient PA 1	Inpatient PA 2

Abbreviations: APP, advanced practice provider; PA, physician assistant; peds oto, pediatric otolaryngology.

### Surgical Case Management and Inpatient Care

In accordance with guidance from the Centers for Disease Control and Prevention, the American College of Surgeons, and the Illinois Department of Public Health, all nonemergent or elective surgery was canceled starting March 16, 2020, at Rush. From an institutional perspective, this would allow physicians and nurses to focus on the pandemic response. In addition, this would allow operating rooms (ORs) and perioperative spaces to be repurposed during the potential surge of patients with COVID-19 and to conserve personal protective equipment (PPE) and redeploy ventilators to other patient care areas as needed. A departmental surgical prioritization list was developed to classify cases into emergent (next available OR, 3-6 hours) and time critical urgent (1-2 weeks; **Table 2**). When oncologic cases were scheduled, the surgical plan and potential adjuvant treatment implications were carefully reviewed at the weekly multidisciplinary tumor board. Treatment preceded with a focus on complete and efficient care while limiting utilization of inpatient resources, patient length of stay, and potential exposure risks to this immunocompromised population. This process was also in accordance with the American College of Surgeons’“COVID-19 Guidelines for Triage of Cancer Surgery Patients.”^6^ The final list was circulated to the perioperative services leadership to ensure timely access to care for critical cases emerging during this period. All other patients from previously scheduled elective cases were personally contacted by their surgeon to postpone or reschedule these procedures.

**Table 2.** List of Emergent and Time-Critical Urgent Cases During the COVID-19 Crisis for Adult and Pediatric Patients.

Emergent (next available OR)	• Acute airway obstruction • Airway foreign body • Angioedema • Deep space neck abscess with airway involvement • Free flap compromise • Neck hematoma • Necrotizing fasciitis • Orbital hematoma • Penetrating neck trauma • Postoperative hemorrhage with hemodynamic instability • Posttonsillectomy hemorrhage
Emergent (to OR in 3-6 h)	• Acute invasive fungal sinusitis • Acute mastoiditis with complications • Bilateral mandible fracture with instability • Esophageal foreign body • I&D deep neck abscess • Orbital abscess • Orbital fracture with entrapment
Time-critical urgent surgery (1-2 wk)	• Advanced-stage head and neck cancer ablative and reconstructive surgery • Benign skull base/head and neck tumors with significant symptoms or concern for permanent neurologic injury (eg, acoustic neuroma, pituitary adenoma, paraganglioma) • CSF leaks/encephaloceles repair with complications • Maxillofacial trauma • Pending or progressive airway compromise • ENT surgery where delay >1 mo may cause permanent neurosensory deficit (eg, sinus disease causing orbital/skull base compression, chronic ear disease with facial weakness or inner ear erosion) • Other clinical scenarios where delay in care could cause significant compromise of patient health, to be approved by departmental surgery review team

Abbreviations: CSF, cerebrospinal fluid; ENT, ear, nose, and throat; I&D, incision and drainage; OR, operating room.

All urgent and time-critical elective cases were reviewed by the chair, vice chair, and patient safety officer to ensure that they were deemed appropriate for personnel and resource utilization and subsequently forwarded to the institutional surgical leadership for posting. Given the high risk of viral shedding in the upper aerodigestive tract, all patients with mucosal cases involving the nose, paranasal sinuses, nasopharynx, oral cavity, oropharynx, larynx, and hypopharynx would undergo preoperative COVID-19 testing 2 to 3 days prior to surgery. By April 9, POC testing was available in the OR for all surgical cases and would facilitate the ability to perform 2 sequential tests and confirm negative results 2 to 3 days apart prior to surgery. This was deemed mandatory for all upper aerodigestive tract cases with high risk of aerosolization. Despite negative test results, all surgeons, anesthesia, and staff would wear N95 respirators in addition to standard PPE for invasive mucosal cases with high risk of aerosolization. PPE recommendations were reviewed daily; video demonstrations were created and posted for hospital-wide access; and provider simulation of PPE donning and doffing was offered. Prior to clustering of cases in the community, our department adjusted PPE recommendations and endoscopy indications for inpatient consultations in anticipation of unrecognized admitted cases. Rounding teams were split, and personnel limitations were created for patient interactions to mitigate potential exposures.

### Tracheostomy Protocol Development

The department also developed a tracheostomy protocol in conjunction with key stakeholders across the institution, including general surgery, critical care, anesthesia, infection control, OR nursing, and respiratory therapy. This was implemented on April 1, 2020, to provide a standardized care pathway, maintenance of infection control, safety of providers, and optimization of patient care outcomes for intubated patients with COVID-19. Percutaneous and bedside/OR open tracheostomy was part of the care pathway, dependent on patient factors and provider considerations. Given the high risk of aerosolization in these potentially positive cases of COVID-19, a specific type of powered air-purifying respirator, termed controlled air-purifying respirator (CAPR; MAXAIR), was utilized. The CAPR integrates the motor, fan, filter, and controlled air flow mechanism directly in a lightweight helmet, thus eliminating the need for the breathing tube connected to a belt-mounted unit. This minimizes the need for rigorous cleaning procedures required with the powered air-purifying respirator and improves physician maneuverability during the procedure. The Rush University Medical Center tracheostomy protocol is available as **Table 3**.

**Table 3.** Rush University Medical Center COVID-19 Tracheostomy Protocol.

Background	Due to the COVID-19 pandemic, a dedicated tracheostomy protocol is being implemented for the purposes of a standardized care pathway, maintenance of infection control, provider safety, and optimization of patient care outcomes.
Protocol development	Coordination and development of the protocol done after consultation with representatives from otolaryngology, general surgery, critical care, anesthesia, infection control, OR nursing, and respiratory therapy. Additional literature review of SARS experience conducted by team. Unpublished data from Wuhan hospitals reviewed.
Indications for surgery	Currently there is no available evidence to support early or aggressive tracheostomy for patients with COVID-19. In fact, early anecdotal evidence from China demonstrates a very low rate of tracheostomy overall in patients with COVID-19. Concerns about the procedure center on intra- and postoperative infection control, care provider safety, post–hospital care setting availability (LTAC), patient safety related to prone positioning, and ultimate effect on mortality outcomes. Therefore, initial protocols will focus on limited consideration of tracheostomy after careful individual patient consideration. • Mechanical ventilation >14-21 d • FiO_2_ <50% • PEEP <8-10 • PIP < 30 • Not requiring high-dose vasoactive agent and/or >1 vasopressor • Absence of uncontrolled dysrhythmia • Absence of severe acidosis • INR <1.5 • Platelets >100k • No anatomic contraindications • Availability of recommended PPE • COVID-19 PCR testing that is negative 2 times before surgery. If testing is positive, surgical and medical teams will discuss risks and benefits of surgery, taking into account all medical, safety, and infection control factors.
Setting/technique	• Initial technique consideration will be to perform percutaneous tracheostomy. • If contraindicated medically or due to other limitations, open technique will be performed. • Bedside technique vs OR after consideration of medical status, including COVID-19 testing.
Staff (bedside): percutaneous tracheostomy	• Attending physician • Resident/fellow physician • Unit nurse inside room • Clean RT inside room for postoperative care
Staff (bedside): open tracheostomy	• Anesthesia • Attending surgeon • Resident surgeon • OR scrub nurse in room • Clean nurse unit nurse outside room • Clean RT outside room for postoperative care
Staff (OR): open tracheostomy	• Anesthesia • Attending surgeon • Resident surgeon • OR scrub nurse in room • Circulating RN in room
Preoperative preparation	• Overhead lights and portable headlight inside hood • Neck extension with shoulder roll • Paralyzed, 100% FiO_2_ • Inject site with 1% lidocaine with 1:100,000 epinephrine • Neck: sterile preparation with face exposed for ETT access • Enhanced PPE: surgical gowns, double gloves, PAPR hoods, shoe covers • Tracheostomy tray, Bovie machine and equipment, OR pack
Intraoperative considerations	• FiO_2_ <30% when incising trachea • Ventilation held when cuff is deflated or when incising trachea • Consider Steri (1010) drape coverage when incising trachea
Postoperative respiratory care	• HME > T-piece > tracheostomy collar • T-piece preferred over tracheostomy collar • Inline suction only • Maintain cuff pressure 25-30 cm H_2_O • Minimize bronchial hygiene: no HyperSal • No prone position
Transfers from outside hospitals	Transfers with “reason for transfer: tracheostomy in COVID-19” will be accepted from outside hospitals only if they meet the indications for surgery after consultation with the surgeon and ICU attending.

Abbreviations: ETT, endotracheal tube; HME, heat moisture exchanger; ICU, intensive care unit; INR, international normalized ratio; LTAC, long-term acute care; OR, operating room; PAPR, powered air-purifying respirator; PCR, polymerase chain reaction; PEEP, positive end expiratory pressure; PIP, peak inspiratory pressure; PPE, personal protective equipment; SARS, severe acute respiratory syndrome; RN, registered nurse; RT, respiratory therapy.

### Ambulatory Practice Management

By March 1, 2020, all patients presenting to the ambulatory sites, including the main campus and 3 regional sites, were thoroughly screened for symptoms and foreign travel. With progressive community outbreak, this screening was no longer considered reliable. By mid-March, all elective in-person clinic visits and office procedures were gradually converted to virtual video or telephone visits for new and established patients to maintain continuity of patient care. All physicians reviewed their upcoming schedules and marked appointments to be rescheduled, converted to a telehealth visit, or seen in person (if critical). During the baseline week prior to implementation, 730 in-person office visits were conducted at the main campus and regional sites. After a 3-week transition period with conversion to digital video and telephone visits, 297 total visits were performed, including 43 in person (14.5%), 97 by telephone (32.7%), and 157 by video (52.9%). In-person outpatient visits were available to patients deemed important for urgent examination, especially for the vulnerable head and neck cancer population or patients in the immediate postoperative period. Audiology services were significantly curtailed, with provision only when testing outcome could significantly affect care decisions, such as sudden sensorineural hearing loss.

Prior to conversion to the virtual platform and continued throughout were strategies to protect providers and support and administrative staff. These included twice-daily symptom and temperature checks, spacing of work stations, social distancing of breakroom activities, staggering of examination room utilization to allow for cleaning, movement of high-risk employees (immunosuppression, comorbidities) away from patient-facing interactions, and frequent review of PPE and protocols for any patients suspected of having COVID-19. Additionally, through the institutional command center structure, select staff volunteered and were reassigned to other clinical areas as needs for COVID-19–related care increased.

With a significant drop in outpatient clinic volume (40% of baseline), provider schedules and templates were consolidated to our main campus with focused access at regional sites to more effectively utilize clinic personnel and to limit foot traffic to clinical areas. Upper airway endoscopy was performed only when deemed absolutely necessary to guide clinical decision making. Full PPE with N95 respirator was utilized for endoscopy, irrespective of the COVID-19 status of the patient.

Clinical providers were able to successfully engage patients via the video platform for most elective and many urgent issues. The initial screening allowed for subsequent in-person visit for pressing urgent matters as needed. With more widespread availability of COVID-19 testing at the POC, within 1 month of implementation of the virtual platform, the faculty practice has now designated 2 off-sites, in the city (Rush South Loop) and a suburb (Rush Oak Brook), as “safe sites” to allow for in-person evaluations for urgent patient care issues. These patients undergo COVID-19 symptom screening at the time of appointment scheduling, 24 hours prior to appointment, and on the day of appointment. All ENT patients also undergo POC testing for COVID-19 and are evaluated in person if testing is negative. This strategy will allow for more optimal management of urgent patient issues while maintaining rigorous safeguards for provider and patient safety. Several email communications were sent to our entire patient base and referring physicians, informing them of their options for care and consultations during the pandemic.

## Residency Program/Education

The substantial decrease in surgical cases and outpatient volume has adversely affected the overall training opportunity for residents. However, the overall engagement into a departmental and institutional response to an evolving global pandemic provided an unparalleled opportunity to engage trainees in a system-based practice format for disaster management. As mentioned, all residents were designated to teams A and B in conjunction with the faculty. One senior resident would lead each resident team and provide nighttime backup coverage to the junior residents on call. The off-call team would not be required to be on-site to reduce potential exposure to patients at the institution and the other team. The postgraduate year 1 and 2 residents (PGY-1 and PGY-2) were also available to assist in the ED and ICU setting as required during the surge plans.

All teaching sessions, including lectures, morbidity and mortality conference, and tumor board, were transitioned to a virtual format via the WebEx platform. Over the initial month, weekly faculty/resident sessions were conducted to communicate the reorganized departmental structure, review newly implemented clinical and OR policies, and address provider concerns. These educational sessions integrated review of the most recent literature and recommendations regarding COVID-19 diagnosis, treatment, safety, and disease course to ensure dissemination of the highest available scientific evidence. These sessions also served to promote engagement, to allay anxiety due to the rapid changes, and to build camaraderie among the team members. Quality initiative projects were also implemented to support the overall organizational effort for COVID-19. Simulation training was conducted for faculty, residents, and nurses to guide proper donning and doffing of CAPR for airway procedures and proper technique for nasal and nasopharyngeal swabbing for COVID-19 testing.

On March 27, 2020, the institution declared a stage 3 pandemic status under the existing extraordinary circumstances policy of the ACGME (Accreditation Council for Graduate Medical Education) with a 30-day expiration to be reconsidered for extension at that time. Rush was the first institution in the Chicago region to make this declaration due to the high volume of patients with COVID-19 being treated at the medical center. This declaration allowed for great flexibility in the training environment—most notably, the redeployment of residents to the COVID-19 surge units. All ACGME program requirements were temporarily waived, with the exception of PPE resources/training, supervision, and work hour requirements. During the initial 3 weeks, 4 otolaryngology residents were deployed to a surge unit in the ED with the purpose of providing direct otolaryngology-related care to offload the ED staff to primarily care for patients with COVID-19. This need quickly reduced and shifted toward inpatient needs after the first week of the patient surge. Critical care platforms soon became the most utilized resource across the institution. Medical specialty residents were fully utilized on inpatient hospital medicine units and existing medical critical care units. The hospital quickly converted additional surge ICU beds that were staffed by surgical residents under supervision by a critical care attending. Two otolaryngology residents have participated in 1-week rotations on these units to date.

## Research Efforts

Research remains a core component of our academic mission. At the time of the pandemic, the department had more than a dozen head and neck oncology, rhinology, sleep, and otology clinical trials in different phases of progression. To mitigate risks to patients, all in-person visits were postponed and follow-up transitioned to telephone visits. New clinical trials and new patient enrollment were temporarily halted until stabilization of the pandemic and to allow providers to focus on COVID-19–related efforts. Multiple retrospective and prospective clinical studies were designed in close collaboration between departmental faculty and residents to investigate the impact of coronavirus on otolaryngology care. Specific efforts include investigations into smell and taste dysfunction in COVID-19 as well as detailed analysis of innovative workflow changes involving POC COVID-19 testing. Additionally, results of simulation-based training initiatives were formalized and submitted for publication.

## Discussion

The evolving pandemic has challenged the provision of otolaryngology care globally in an unmatched manner. The elective nature of much of our specialty has resulted in a drastic reduction of the ambulatory clinic and operative volumes so that personnel and resources can be appropriately diverted to support providers on the frontline of battling the COVID-19 pandemic. However, this also represents a unique opportunity to recalibrate the clinical and academic goals of departments to align with the mission of their home institutions and local communities to meet the greater needs of the population in this extraordinary time. Most academic medical centers represent complex matrixed organizations where change management can be difficult and often slow due to multiple stakeholders and complex rules and regulations. Within 3 weeks, the entire health care system was able to transform the physical space and repurpose personnel to meet the challenges of the emerging pandemic. In parallel, the department was reorganized with a complete shift in focus to continue to provide essential ENT services but also meet the institutional needs in the broader context of the COVID-19 pandemic. The key departmental initiatives included (1) reorganization of the department into a 2-team structure for call and surge redeployment, (2) cessation of elective surgery and creation of an emergent and time-critical urgent case list, (3) transition of in-person visits to the virtual video platform and creation of safe regional sites, (4) adaptation of a web-based platform for weekly departmental meetings and educational conferences, (5) maintenance of patient engagement with close frequent communication to existing and potentially new patients on availability for telehealth and in-person options, and (6) refocus of research efforts to COVID-19–related projects.

Despite the rapid nature of the required changes, the overall engagement of the faculty, trainees, and staff has remained high as they have shifted their efforts to meet the new demands and challenges posed by the health crisis. This magnitude of change is possible only through continuous involvement and input from all stakeholders and constant communication through formal meetings and informal touch points. As it stands, this study is largely process driven and lacks data on specific outcomes of the implemented changes. Five studies are currently underway to assess the impact of the myriad changes, including the aforementioned simulation projects and POC testing in the operative and ambulatory environment. Two manuscripts have already been submitted to *Otolaryngology–Head and Neck Surgery*: one on simulation training to guide proper donning and doffing of CAPR for airway procedures and the other on the impact of POC testing in the OR.

## Conclusion

This article outlines the implementation strategy of changes required to the departmental structure and delivery of care during the emerging pandemic. The changes implemented weighed the competing priorities of the departmental provision of otolaryngology care with the institutional priorities for pandemic management, with the prime directive being to preserve the health and safety of the workforce. This can serve as a template for other academic departments and group practices nationally and internationally to meet these new unmet challenges, since the infection will peak at differing times in various parts of the world and thus disrupt delivery of care in an asynchronous manner.
